# RETRACTION: Artificial Dermatitis Versus Artificial Intelligence: Detecting Etiology With Google Lens

**DOI:** 10.1111/srt.70340

**Published:** 2026-02-12

**RETRACTION**: E. Ayhan and Y. Alagöz, “Artificial Dermatitis Versus Artificial Intelligence: Detecting Etiology with Google Lens,” *Skin Research and Technology* 30, no. 6 (2024): e13725, https://doi.org/10.1111/srt.13725.

The above article, published online on 17 June 2024 in Wiley Online Library (wileyonlinelibrary.com), has been retracted by agreement between *Skin Research and Technology*; and John Wiley & Sons Ltd. The article was accidentally published as a wrong manuscript type and accepted for publication following an insufficient peer review process that does not meet the standards of the journal's peer review policy. Furthermore, the article contains images sourced from the internet without authorization for use. Accordingly, the article has been retracted. The authors disagree with this decision.

